# RF-SOI Low-Noise Amplifier Using RC Feedback and Series Inductive-Peaking Techniques for 5G New Radio Application

**DOI:** 10.3390/s23135808

**Published:** 2023-06-22

**Authors:** Min-Su Kim, Sang-Sun Yoo

**Affiliations:** 1 Department of Information and Electronic Engineering, Mokpo National University, Muan 58554, Republic of Korea; msmy970@mnu.ac.kr; 2 Department of Smart Automobile, Pyeongtaek University, Pyeongtaek-si 450-701, Republic of Korea

**Keywords:** RF-SOI (radio frequency silicon-on-insulator), low-noise amplifier (LNA), RC feedback, inductive peaking, 5G, New Radio (NR) frequency band

## Abstract

This paper presents a low-noise amplifier (LNA) with an integrated input and output matching network designed using RF-SOI technology. This LNA was designed with a resistive feedback topology and an inductive peaking technology to provide 600 MHz of bandwidth in the N79 band (4.4 GHz to 5.0 GHz). Generally, the resistive feedback structure used in broadband applications allows the input and output impedance to be made to satisfy the broadband conditions through low-impedance feedback. However, feedback impedance for excessive broadband characteristics can degrade the noise performance as a consequence. To achieve a better noise performance for a bandwidth of 600 MHz, the paper provided an optimized noise performance by selecting the feedback resistor value optimized for the N79 band. Additionally, an inductive peaking technique was applied to the designed low-noise amplifier to achieve a better optimized output matching network. The designed low-noise amplifier simulated a gain of 20.68 dB and 19.94 dB from 4.4 to 5.0 GHz, with noise figures of 1.57 dB and 1.73 dB, respectively. The input and output matching networks were also integrated, and the power consumption was designed to be 9.95 mA at a supply voltage of 1.2 V.

## 1. Introduction

For the efficient utilization of the frequency spectrum, the 5G new radio frequency bands (3.3 to 4.2 GHz of N77, and 4.4 to 5.0 GHz of N79, respectively) has recently received a lot of attention, and various studies for mobile communication are being conducted for this purpose. The broadband low-noise amplifier (LNA) was designed using various design methods, and in particular, broadband low-noise amplifiers using feedback techniques have been commonly used [1,2,3,4]. Such feedback techniques have the advantage of enabling the broadband matching of the required impedance for the broadband’s performance. However, it is difficult to achieve an optimized performance due to the trade-off relationship between the gain and noise performance. In addition to these methods, various design techniques, such as series peaking and gm-boosting are currently being assessed as broadband techniques [5,6].

**Figure 1:**
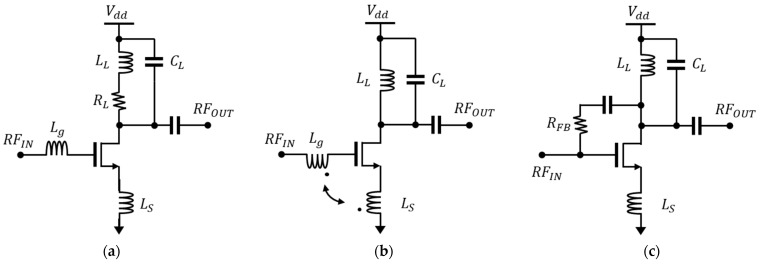
The broadband LNA topologies. (**a**) Inductively degenerated LNA with shunt peaking, (**b**) gm-boosted LNA, and (**c**) resistive-feedback LNA.

Figure 1 shows various broadband techniques. The shunt peaking in Figure 1a adds a series resistor and inductor to compensate for the impedance drop due to the parasitic capacitance at the output [5]. This helps in reducing the rolling down of the output frequency response resulting in a broadband characteristic. However, adding a resistor creates voltage headroom, which is the limiting factor for high-voltage operations. Figure 1b displays a method using gm-boosting, which has the advantage of minimizing the sensitivity to the bandwidth and input current noise [6]. However, using larger transistors to reduce input impedance results in increased power consumption. Furthermore, the increase in parasitic capacitance from larger transistors necessitates the inclusion of additional compensation circuits to minimize its effects, thereby increasing circuit complexity. 

Figure 1c shows a commonly used structure with feedback, where matching the input impedance can be designed using the appropriate feedback resistor (*R_FB_*) value [6]. However, this feedback noise cannot be arbitrarily removed, and is amplified as a result, causing the increase in the output noise, along with the occurrence of performance degradation.

In this paper, a low-noise amplifier design using an optimum RC feedback and inductive peaking technique is presented for the N79 band, covering a frequency range of 4.4 GHz to 5.0 GHz using the RF Silicon on Insulator (RF-SOI) process. To design the input and output matching network for N79 band applications, the optimal resistor value of resistive feedback was selected and subsequently optimized. In addition, an inductive peaking technique was added for the output matching network to ultimately achieve a better matching network and an optimized low-noise amplifier design. Furthermore, the proposed low-noise amplifier is designed with integrated matching networks for both input and output, enabling high integration without the need for additional external matching circuits. Section 2 presents the design methodology using resistive feedback, and Section 3 explains the inductive peaking techniques used in this paper, following which the simulation results and conclusions are presented.

## 2. Resistive Feedback for the Wide Input Matching Network

**Figure 2:**
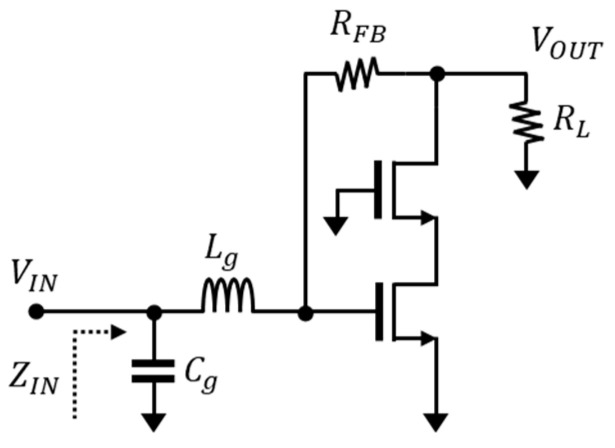
Simplified schematic of a Cascode low-noise amplifier.

The resistive feedback technique has been commonly used for broadband low-noise amplifiers. However, although it can have a wideband performance according to the feedback structure, the output noise is also fed back, and is in a trade-off relationship that degrades the noise of the low-noise amplifier. Therefore, careful design consideration is necessary. Figure 2 shows a simplified circuit diagram of a Cascode low-noise amplifier with resistive feedback. Cascode LNAs have been widely used for their high gain and low-noise characteristics, including inductor (*L_g_*) and capacitor (*C_g_*) for matching. However, due to its narrowband characteristics, it is challenging to apply this to the broadband, meaning resistive feedback must be used to design the broadband. 

**Figure 3:**
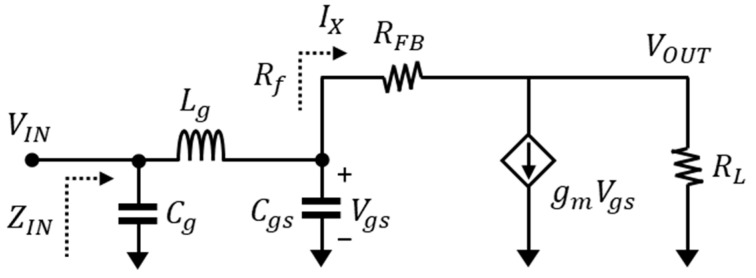
Simplified small-signal model of a resistive feedback common-source amplifier.

Figure 3 illustrates an equivalent small-signal model using a common-source amplifier for the simplification of analysis. In this case, the input impedance of the LNA with feedback, including *R_FB_*, can be derived as follows.

(1) $$I_{X}=\frac{V_{gs}-V_{out}}{R_{FB}},\;I_{X}=g_{m}V_{gs}+\frac{V_{out}}{R_{L}}$$

By expressing the current flowing into the *R_FB_* resistor as *I_x_*, the above equation can be obtained. *C_gs_* and *g_m_* are the gate-source capacitance and the transconductance of the transistor, respectively, and *R_L_* is the load resistance. Using these equations, we can represent the impedance observed by the feedback as follows:

(2) $$R_{f}=\frac{V_{gs}}{Ix}=\frac{R_{FB}+R_{L}}{1+g_{m}R_{L}}$$

In this case, the intrinsic gate-drain capacitance *C_gd_* was ignored. The input impedance changes due to the feedback current *I_x_*, and this can be expressed as follows:

(3) $$\begin{array}{c} Z_{IN,w\;FB}=\frac{1}{sC_{g}}\;||\;\left[sL_{S}+\left(\frac{1}{sC_{gs}}||R_{f}\right)\right]\\ =\frac{1}{sC_{g}}||\frac{s^{2}L_{g}C_{gs}R_{f}+sL_{g}+R_{f}}{sC_{gs}R_{f}+1}=\frac{s^{2}L_{g}C_{gs}R_{f}+sL_{g}+R_{f}}{s^{3}L_{g}C_{gs}C_{g}R_{f}+s^{2}L_{g}C_{g}+sR_{f}\left(C_{g}+C_{gs}\right)+1} \end{array}$$

**Figure 4:**
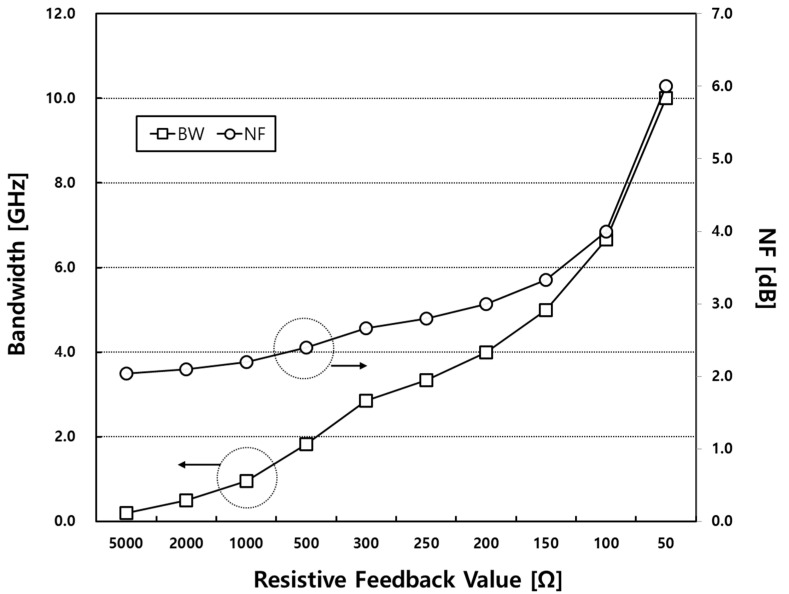
The simulated bandwidth and noise figure according to the resistive feedback value.

To achieve a perfect matching condition of *Z_in_* = *Z_S_*, it has thus been commonly assumed that *R_f_* = *Z_S_* and *C_g_* = *C_gs_*. By setting *C_g_* equal to *C_gs_*, which minimizes the parasitic capacitance present in the input, and having *R_f_* and *Z_S_* with an equal impedance, the best matching condition can thereby be achieved. Using the given equations, we can express $\left|S_{11}\right|$ in terms of *Z_in_* and *Z_S_*, as follows:

(4) $$\left|S_{11}\right|=\left|\frac{Z_{in}-Z_{S}}{Z_{in}+Z_{S}}\right|=\left|\frac{-s^{3}L_{g}Z_{S}^{2}C_{gs}^{2}+s\left[L_{g}-Z_{S}^{2}2C_{gs}\right]}{s^{3}L_{g}Z_{S}^{2}C_{gs}^{2}+s^{2}L_{g}Z_{S}2C_{gs}+s\left[L_{g}+Z_{S}^{2}2C_{gs}\right]+2Z_{S}}\right|$$

where the response of $\left|S_{11}\right|$ includes two zero points. *ꞷ*_02_ is as follows [7]:

(5) $$\omega_{02}=\sqrt{\frac{2}{L_{g}C_{gs}}-\frac{1}{Z_{S}^{2}C_{gs}^{2}}}=\frac{1}{\sqrt{L_{g}C_{gs}}}$$

where according to the condition of *L_g_* = *R_f_*^2^*C_gs_* and *C_g_* = *C_gs_*, the input matching network has a symmetrical third-order, ladder-type, low-pass filter characteristic. In this case, the frequency of *ꞷ*_02_ can be adjusted using the inductance of *L_g_* and the capacitance of *C_gs_* to provide a matching condition of −10 dB or more, thereby making it possible to design a broadband matching network. However, in the case of a feedback resistor, a low impedance feedback structure cannot be applied due to the trade-off relationship between the bandwidth and the noise figure (*NF*). Therefore, optimization between the desired bandwidth for the frequency range extension and the targeted *NF* becomes essential. *NF* can be represented using a model of the common-source amplifier with resistive feedback, which is as follows [8]:

(6) $$NF\approx 1+\frac{4Rs}{R_{FB}}+\gamma +\gamma g_{m}R_{s}$$

where γ represents the excess noise coefficient of the MOSFET. As shown in Equation (6), if the *R_FB_* has a low value, the *NF* performance will degrade as a consequence. Figure 4 is a simulation result showing the relationship between the bandwidth and the *NF* according to the feedback resistor value. As the resistor value of *R_FB_* decreases, the bandwidth of the input matching increases as a result. However, in the case where the NF performance degrades, the feedback resistor value used for the desired broadband matching should be matched using the optimal value.

## 3. Inductive Peaking for the Wide Output Matching Network

**Figure 5:**
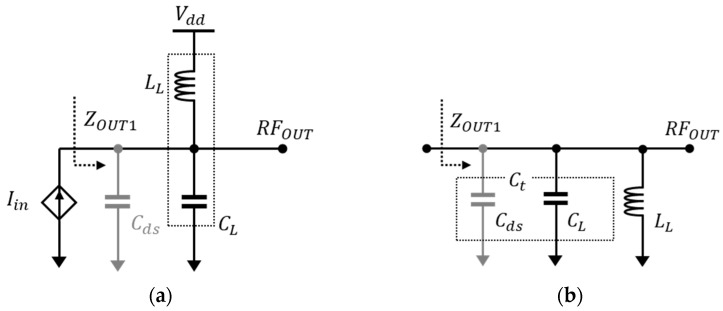
Conventional (**a**) output matching network and (**b**) simplification.

Figure 5 shows the output matching network for a conventional low-noise amplifier. Here, *L_L_* and *C_L_* represent the frequency response characteristics of the bandpass filter, and *C_ds_* is the parasitic source-drain capacitance generated in the intrinsic transistor, respectively. *C_ds_* is combined with the load *C_L_*, and together with *L_L_*, has a resonance frequency $\omega_{0}$. The impedance of the output matching network can be expressed as:

(7) $$Z_{OUT1}=\frac{1}{sC_{t}}\;||\;sL_{L}=\frac{sL_{L}}{s^{2}C_{t}L_{L}+1}$$

As shown in Equation (7), the output impedance can be decreased depending on the *C_t_* of the matching circuit, and due to the capacitance of *C_ds_*, it will thereby have a more abrupt frequency response characteristic. *C_t_* represents the total capacitance value of *C_ds_* and *C_L_*.

**Figure 6:**
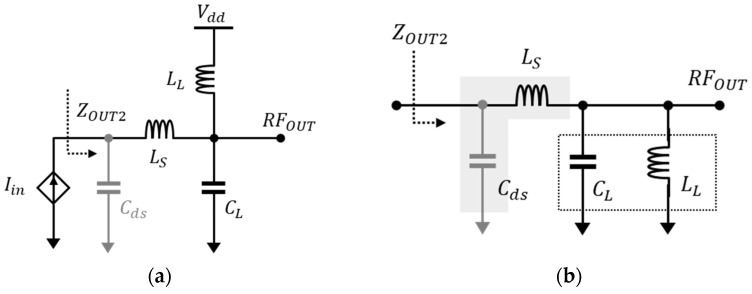
The (**a**) output matching network and (**b**) simplification.

For the wide output matching network design, adding a series inductor *L_s_* to the output matching network has been considered, as shown in Figure 6. The addition of a series *L_s_* could be able to compensate for the frequency response degradation caused by the *C_ds_* of the intrinsic transistor. This inductive peaking technique has been expanded to the resonance frequencies of *L_s_* and *C_ds_*, as well as to *C_L_* and *L_L_*, thereby allowing it to operate as a wide matching network. The output impedance can be expressed as:

(8) $$Z_{OUT2}=\left(\frac{1}{sC_{ds}}\right)\;||\;\left(\;sL_{s}+\left[\frac{1}{sC_{L}}\;||\;sL_{L}\right]\right)=\frac{s^{3}C_{L}L_{L}L_{S}+s\left(L_{S}+L_{L}\right)}{s^{4}C_{ds}C_{L}L_{L}L_{S}+s^{2}\left(C_{ds}L_{S}+C_{ds}L_{L}+C_{L}L_{L}\right)+1}$$

When making the imaginary part of the output impedance zero, we obtain the following Equation (9), which is the characteristic equation involving the frequency ꞷ as follows:

(9) $$\omega^{4}L_{L}C_{L}L_{S}C_{ds}-\omega^{2}\left(L_{S}C_{ds}+L_{L}C_{ds}+L_{L}C_{L}\right)+1=0$$

Equation (9) can be simplified to the product of the sum of roots *S* and the root *P*, respectively, as previously shown in [9].

(10) $$S=\omega_{1}{}^{2}+\omega_{2}{}^{2}=\frac{C_{ds}L_{S}+C_{ds}L_{L}+C_{L}L_{L}}{C_{ds}C_{L}L_{L}L_{S}}=\frac{1}{C_{L}L_{L}}+\frac{1}{C_{L}L_{S}}+\frac{1}{C_{ds}L_{S}}$$

(11) $$P=\omega_{1}{}^{2}\omega_{2}{}^{2}=\frac{1}{C_{ds}C_{L}L_{L}L_{S}}$$

Using Equations (10) and (11), we can determine three resonance frequencies through the output matching network: $\omega_{1,\;2}$, and the average of the two operating radian frequencies, which hence referred to as $\omega_{3}$. The $\omega_{3}$ resonance frequency can be determined using the ratio of *L_S_* to *L_L_* and *C_L_* to *C_ds_*, respectively, and the network should be designed with an optimal matching value to achieve the desired performance.

## 4. Low-Noise Amplifier Design for the NR 79 Band

**Figure 7:**
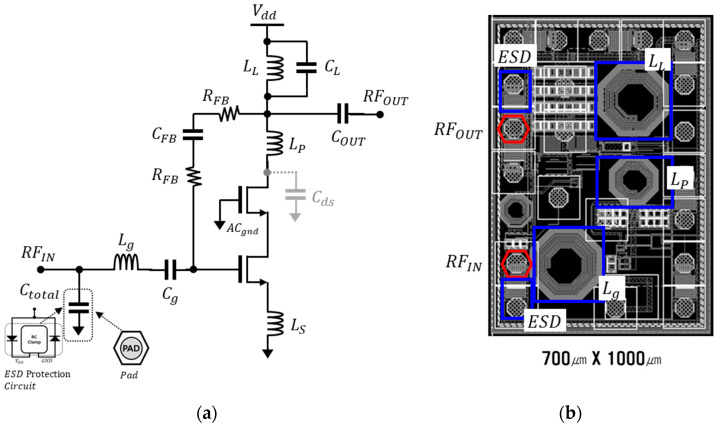
The (**a**) Schematic and (**b**) layout of the designed LNA.

Figure 7a shows a Cascode low-noise amplifier, which uses the resistive feedback structure and an inductive peaking technique for the N79 band. The decoupling capacitors *C_g_*, *C_FB_*, and *C_out_*, were all added to the design. *C_total_* is an equivalent capacitance that represents the combination of parasitic capacitance from the Electrostatic Discharge (ESD) protection circuit and bump pad and was designed to be included in the input matching network. In the previous section, *C_total_*, similar to *C_g_* in Figure 5, is a parameter that needs to be taken into consideration, as it can unexpectedly affect the matching conditions. 

In a real design, resistors are accompanied with parasitic parallel capacitors that are generated in the layout. This parasitic parallel capacitor is a capacitor between the metal line for connection and the substrate, and the larger the resistance, the larger the parasitic capacitor that is formed. Therefore, to achieve the same effect for the output and input in the feedback structure, the resistive feedback (*R_FB_*) was symmetrically applied with a decoupling capacitor to achieve the required resistance for the resistive feedback structure. *C_ds_* represents the capacitance generated at the drain of the common-gate transistor in the Cascode amplifier. As *C_ds_* performs a function that causes a faster rolling down depending on the frequency in the general output matching network, *L_P_* was added as a compensation. Then, the output matching with the third pole was designed with *L_L_* and *C_L_*. Figure 7b represents the chip layout of the designed LNA, which was designed at a size of 700 µm × 1000 µm and includes a ESD protection circuit for the input and output, as well as three matching inductors for impedance matching. Furthermore, the layout includes ground bumps for the AC grounding of the common-gate stage.

**Figure 8:**
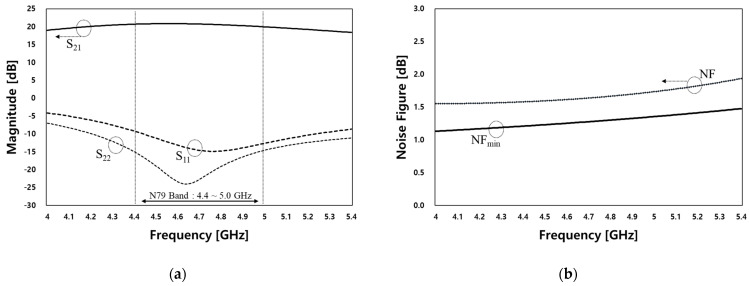
Simulated (**a**) S_21_, S_11_, and S_22_, and (**b**) NF and NFmin performance.

The designed LNA was implemented using the GlobalFoundries RF-SOI 90 nm process. The widths of the two transistors were designed to be 128 µm, and the sum of the synthetic resistor of feedback resistors was 7 k ohm to achieve the desired gain and NF performance, respectively. A small degeneration inductor, Ls, was added to the 120 pH inductor to obtain better matching conditions. Figure 8 shows the simulation results of the LNA for the N79 band. The LNA consumes a current of 9.95 mA at a 1.2 V supply voltage. Figure 8a shows the forward gain S_21_, input, and output reflection coefficients (S_11_ and S_22_, respectively). Figure 8b presents the simulated results of the NF and NF_min_ up to 5.4 GHz. The LNA exhibited a gain of 20.68 dB at 4.4 GHz with the input and output reflection coefficients of −9.24 dB and −15 dB, respectively. At 5.0 GHz, it exhibited a gain of 19.94 dB, with the input and output reflection coefficients of −12.6 dB and −14.6 dB, respectively.

The simulated NF was determined to be 1.57 dB and 1.73 dB, with NF_min_ values of 1.2 dB and 1.32 dB at 4.4 GHz and 5.0 GHz, respectively. This paper applied resistive feedback for designing an LNA operating in the N79 band. Although the designed LNA achieved a bandwidth of 600 MHz, it was designed with a performance difference of around 0.38 dB from the optimal low-noise performance, NF_min_, as shown in Figure 7b. However, it has the advantage of including an internal input/output matching network, which shows an excellent performance from the point of view of the NF. This is because the inductor used in typical chip internal matching networks may cause degradation in the noise performance with a relatively low Q value but may still have an advantage in terms of size in a circuit configuration for an external matching circuit. 

**Figure 9:**
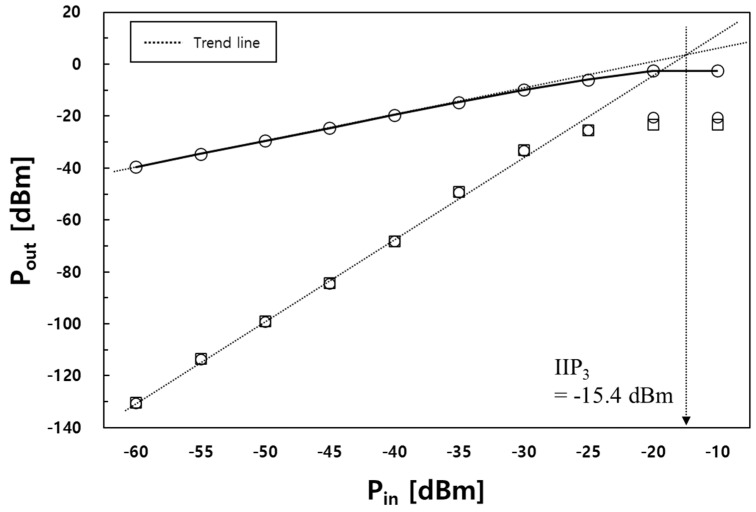
Simulated output power with respect to the input power.

In Figure 9, the input third-order intercept point (IIP_3_) was simulated to be −15.4 dBm at 1 MHz tone spacing. The LNA using feedback generally exhibited an improved linearity compared to the conventional LNAs, as the feedback had a greater effect on the high power obtained from the input. 

A summary of the results is provided in Table 1. Generally, LNAs using resistive feedback possess wide broadband characteristics. However, this paper presents an optimized LNA design for the N79 band using appropriate resistive feedback and inductive peaking techniques, rather than a typical broadband LNA design. Therefore, it was compared with previous studies which assessed in the 0.2 to 5 GHz band. As shown in Table 1, the designed LNA satisfies the bandwidth requirement for the N79 band and exhibits excellent performance in terms of the NF and IIP_3_. 

## 5. Conclusions

This paper presents the design of a low-noise amplifier using the RF-SOI process. The designed low-noise amplifier applied resistive feedback and an inductive peaking technique for the N79 band operation. Although the additional inductive peaking technique includes an increase in the chip size, this paper integrated the matching network that needs to be used externally to eliminate the additional external matching components. In addition, to minimize the occurrence of errors between the design and measurements, circuit design was performed with EM-based simulation results and the PEX-based model. This low-noise amplifier was designed and simulated to achieve an optimal performance for a bandwidth of 600 MHz, and these broadband techniques can be appropriately applied to various low-noise amplifier designs. 

## Figures and Tables

**Table 1:** LNA performance summary and comparison.

Ref.	Technology	Frequency [GHz]	S_11_/S_22_ [dB]	Gain [dB]	NF [dB]	IIP_3_ [dBm]	Area [mm^2^]
[10]	180 nm CMOS	3–5	<−10.5/-	16	1.8	−9	0.63
[11]	65 nm CMOS	0.2–5	-/-	15.6	<3.5	>0	0.009
[12]	180 nm RFSOI	5	−33/−28	11	0.95	5	0.29
	180 nm RFSOI	5	−22/−28	9.3	1.9	6.5	0.29
This work *	90 nm RFSOI	4.4–5.0	−9.4/−15	20.6–19.9	1.57–1.73	−15.4	0.7

* Simulation results.

## Data Availability

Not applicable.
